# Endocrinologist-led glucose management in the emergency ICU: a retrospective before-after study

**DOI:** 10.3389/fendo.2026.1790977

**Published:** 2026-04-20

**Authors:** Long Zhang, Zhang-Sheng Zhao, Chun-Xia Gu, You-Li Ma, Xiao-Fang Yu

**Affiliations:** 1 Department of Endocrinology, The Affiliated LihuiLi Hospital of Ningbo University, Ningbo, Zhejiang, China; 2 Transfusion Medicine Center, Apheresis and Transfusion Therapy Center, The Affiliated LihuiLi Hospital of Ningbo University, Ningbo, Zhejiang, China; 3 Department of Neurology, The Affiliated LihuiLi Hospital of Ningbo University, Ningbo, Zhejiang, China

**Keywords:** continuous glucose monitoring, critical illness, endocrinologist-led management, glycemic control, insulin pump, intensive care unit

## Abstract

**Objective:**

To evaluate the effectiveness and safety of an endocrinologist-led glucose management (ELGM) model integrating real-time continuous glucose monitoring (RT-CGM) and continuous insulin infusion therapy, compared with conventional glucose management in critically ill patients admitted to the emergency intensive care unit (EICU).

**Methods:**

In this single-center, retrospective before-after study, adult EICU patients with stays ≥24 hours were included. Patients admitted in 2023 received conventional glucose management (control), whereas those admitted in 2024 were managed using the ELGM model. Endocrinologists served as primary decision-makers, supported by RT-CGM, point-of-care testing, and continuous insulin infusion. Primary outcomes included mean glucose, glycemic variability, proportion of target-range glucose (3.9 - 10.0 mmol/L), and hypo-/hyperglycemia incidence. Secondary outcomes included nosocomial infection, hyperosmolar hyperglycemic state (HHS), length of stay, ventilation duration, and hospitalization costs.

**Results:**

A total of 1138 patients were analyzed (ELGM 625; Control 513). Compared with conventional management, the ELGM model was associated with lower mean glucose (9.9 vs. 11.1 mmol/L; P< 0.001) and reduced variability (SD 4.2 vs. 4.9 mmol/L; P< 0.001). Target-range readings were higher (61.11% vs. 51.66%; P< 0.001), and severe hyperglycemia was less frequent (7.45% vs. 13.28%; P< 0.001). Severe hypoglycemia did not differ. Nosocomial infection (15.02% vs. 27.68%) and HHS (1.28% vs. 5.65%) were lower (both P< 0.001). Length of stay and costs were similar, while ventilation duration was slightly longer.

**Conclusion:**

An endocrinologist-led glucose management strategy incorporating RT-CGM was associated with improved glycemic metrics and lower rates of severe hyperglycemia and infection, supporting its feasibility in critically ill patients.

## 1 Introduction

Hyperglycemia occurs in up to 80% of patients admitted to the intensive care unit (ICU) (1). Inadequate glycemic control is strongly associated with higher risks of infection, organ failure, and mortality (2–4), and is recognized as a major determinant of poor outcomes in critical illness (5–7). Conventional ICU glucose management typically relies on physicians and nurses applying department-specific experience, with endocrinology consultations sought only when dysglycemia becomes unmanageable. This reactive, consultation-based model is characterized by delayed responses, fragmented management, and limited specialty input, making timely and systematic correction of glycemic abnormalities difficult (8–11).

The introduction of advanced tools such as continuous glucose monitoring (CGM) and insulin infusion systems offers new opportunities for precision glucose management, yet their optimal use requires specialized expertise (12–14). For non-endocrinology staff, interpretation and decision-making remain challenging, which has constrained broader adoption in the ICU setting. Collectively, these limitations hinder the transition toward proactive, standardized, and high-quality glycemic care in critical illness.

Recently, the concept of hospital-wide, multidisciplinary glucose management has emerged, emphasizing specialist leadership and digital support to improve efficiency and safety Building on this framework (15–18), in 2024, our EICU implemented endocrinologist-led glucose management (ELGM) model that combined process optimization, continuous digital monitoring, and proactive intervention. Unlike previous approaches targeting only patients with severe hyperglycemia, the present study evaluated all EICU admissions, aiming to determine the system-wide benefits of this model on glycemic control. By transferring primary responsibility from EICU staff to endocrinology specialists and establishing a closed-loop quality-control system of monitoring, early warning, and intervention, this model was expected to improve both glucose regulation and clinical outcomes. Accordingly, this study sought to validate the effectiveness of the endocrinologist-led glucose management model and to assess its potential value as a scalable strategy for critical care glucose management.

## 2 Materials and methods

### 2.1 Study design and population

This single-center, retrospective, before-and-after study was conducted in the Emergency Intensive Care Unit (EICU) of Ningbo Medical Center Lihuili Hospital. The study aimed to evaluate the effectiveness of an endocrinologist-led glucose management (ELGM) model, which integrates the hospital information system (HIS), real-time continuous glucose monitoring (CGM), and continuous subcutaneous insulin infusion (CSII), compared with the traditional glucose management model in critically ill patients with dysglycemia.

Clinical data were extracted from the electronic medical record (EMR) system, including age, sex, history of diabetes/ketosis acidosis, glycated hemoglobin (HbA1c), glycated serum protein (GSP), 24-hour sequential organ failure assessment (SOFA) score, and principal diseases (cerebral hemorrhage, pneumonia, cerebral infarction, trauma, and myocardial infarction). All data were anonymized before analysis, with removal of all identifiable personal information to ensure privacy protection and non-traceability. In addition, clinically relevant factors that may influence glycemic control during critical illness were collected, including systemic corticosteroid therapy, nutritional support strategy (enteral or parenteral nutrition), and use of continuous renal replacement therapy (CRRT) during the EICU stay.

Beginning in January 2024, the ELGM model was fully implemented for EICU patients. Accordingly, patients were divided into two groups based on the management model received: ELGM group: Patients admitted between January 2024 and December 2024. Control group: Patients admitted between January 2023 and December 2023 who received traditional glucose management.

Inclusion Criteria: Length of EICU stay ≥ 24 hours; Exclusion Criteria: Missing key clinical data exceeding 20%. The study protocol was reviewed and approved by the Institutional Review Board of our hospital (approval number: KY2025SL087-01), with a waiver of written informed consent due to the retrospective design. The study was conducted in accordance with the principles of the Declaration of Helsinki.

### 2.2 Glucose control targets

Although the optimal glucose control target for critically ill patients remains debated, current evidence supports avoiding both severe hyperglycemia and drug-induced hypoglycemia, with recommendations to prevent glucose levels above 10 mmol/L or below 4.4 mmol/L (19–22). In this study, the glucose control target was defined as 7.8 - 10.0 mmol/L.

### 2.3 Glucose management models

#### 2.3.1 ELGM group

Upon EICU admission, all patients underwent point-of-care glucose testing (POCT) every 6 hours (q6h). Patients with a known history of diabetes or those presenting with at least two random glucose measurements ≥ 7.8 mmol/L were routinely equipped with real-time continuous glucose monitoring (RT-CGM), thereby upgrading glucose monitoring from intermittent to continuous surveillance.

In this workflow, POCT served as the reference standard for therapeutic decision-making, whereas RT-CGM was primarily used for continuous trend monitoring and early detection of potential glucose excursions between scheduled POCT measurements. When CGM indicated abnormal trends (e.g., rapid excursions, potential hypoglycemia, or marked hyperglycemia), confirmatory POCT was performed before treatment adjustment. This “POCT-guided, CGM-informed” strategy allowed CGM to function as a decision-support and early-warning tool rather than a direct basis for insulin dose adjustment. Because RT-CGM data alone were not used for insulin dose adjustment, q6h POCT was maintained throughout hospitalization.

A dedicated endocrinology post was established to oversee glucose management in the EICU. Using the hospital information system (HIS) integrated with the CGM data platform, endocrinologists continuously monitored both POCT results and real-time glucose levels and trends of all EICU patients. When glucose values deviated from the predefined target range or unfavorable trends were identified, endocrinologists proactively adjusted treatment regimens directly through the HIS or communicated with on-duty EICU staff via instant messaging tools, enabling immediate intervention without awaiting formal consultation requests.

RT-CGM was routinely applied to obtain continuous glucose data, and continuous subcutaneous insulin infusion (CSII) via insulin pumps was prioritized as the primary insulin delivery strategy, reducing reliance on intravenous insulin infusion when feasible. Therapeutic interventions included subcutaneous insulin administration, intravenous insulin infusion, and CSII via insulin pumps. When intravenous insulin infusion was required, POCT frequency was increased to every 1–2 hours (q1-2h). For patients receiving subcutaneous insulin or CSII, q6h POCT monitoring was continued.

To ensure standardized implementation, a structured standard operating procedure (SOP) was developed to delineate responsibilities between the EICU and endocrinology teams. Regular training sessions were conducted by the endocrinology team for EICU staff, focusing on RT-CGM application, data interpretation, insulin pump management, and troubleshooting, thereby ensuring smooth interdisciplinary collaboration.

#### 2.3.2 Control group (traditional management)

Patients underwent routine q6h POCT. EICU physicians managed dysglycemia based on clinical experience. Glucose management was conducted according to standard EICU practice at that time, with physicians adjusting insulin therapy based on clinical experience rather than a structured protocol. Endocrinology consultations were requested only for patients with difficult-to-control hyperglycemia; the consulting endocrinologist reviewed the electronic medical record and provided written recommendations, which EICU physicians then converted into medical orders for nurses to execute.

Interventions included subcutaneous insulin therapy and intravenous insulin infusion. POCT frequency increased to q1h - q2h when intravenous insulin was administered. Patients often required multiple endocrinology consultations during hospitalization, potentially by different endocrinologists each time.

#### 2.3.3 Glucose-related indicators

For all patients, q6h POCT glucose values were extracted for analysis. If a patient underwent more frequent monitoring (e.g., q1h or q2h), only the q6h glucose measurements were retained, and the additional values were excluded. Because the ELGM model was implemented as a unit-wide workflow intervention affecting the entire EICU rather than individual patients, glycemic indicators were analyzed across all recorded glucose measurements to reflect overall departmental performance. This measurement-level analysis is consistent with quality improvement metrics commonly used in critical care practice, such as time-in-range and hypoglycemia rates.

The mean glucose level and standard deviation (SD) were calculated for each group. Glucose values were further categorized into predefined ranges: 0 - 3.9 mmol/L (Hypoglycemia), 3.9 - 10.0 mmol/L (Normal), 10.0 - 13.9 mmol/L (Level 1 hyperglycemia), 13.9 - 16.7 mmol/L (Level 2 hyperglycemia), and > 16.7 mmol/L (Level 3 hyperglycemia) (23–26), and the proportion of readings within each range was determined.

Patients were also classified according to their most extreme glucose values. For those with hyperglycemia, grouping was based on the highest recorded glucose value; for those with hypoglycemia, grouping was based on the lowest recorded value. The same glucose intervals were used: 0 - 3.9 mmol/L, 3.9–10 mmol/L, 10 - 13.9 mmol/L, 13.9 - 16.7 mmol/L, and > 16.7 mmol/L. For each category, the percentage of glucose measurements within the target range (PGMR) was calculated. PGMR was defined as the proportion of blood glucose readings within 3.9 - 10.0 mmol/L, calculated by:


$$\text{PGMR }=\frac{\text{Readings within }3.9\;-10.0\text{ mmol}/\text{L}}{\text{Total number of glucose measurements}}\times 100\%$$

In addition, the proportions of drug-induced hypoglycemia, severe hypoglycemia (<3.0 mmol/L), and mild hypoglycemia (3.0-3.9 mmol/L) were recorded. Drug-induced hypoglycemia was defined as any blood glucose value< 3.9 mmol/L occurring during treatment with glucose-lowering medications (e.g., insulin or insulin secretagogues). Hypoglycemia clearly attributable to other identifiable causes, such as severe systemic illness, endocrine disorders, interruption of nutritional support, or non-glucose-lowering drugs, was excluded.

### 2.4 Outcome

#### 2.4.1 Primary outcomes

The primary outcomes of this study focused on glycemic control during the EICU stay and included the following parameters:

1. Mean glucose level and glycemic variability, calculated from all retained q6h POCT measurements.
2. Percentage of glucose measurements within the target range (PGMR).
3. Incidence of hyperglycemia and hypoglycemia.

#### 2.4.2 Secondary outcomes

Secondary outcomes included clinically relevant patient-centered endpoints:

1. Incidence of hospital-acquired infection during hospitalization.
2. Incidence of hyperglycemic hyperosmolar state (HHS).
3. Length of stay (LOS) in the EICU.
4. Total hospital length of stay.
5. Total hospitalization cost.
6. Duration of endotracheal intubation.

### 2.5 Statistical analysis

All statistical analyses were performed using Python 3.9. Continuous variables were expressed as mean ± standard deviation (SD) and compared between groups using the independent-samples t test. Given the relatively large sample size, the t test was considered robust to moderate deviations from normality. Categorical variables were expressed as proportions (%) and compared using the χ² test or Fisher’s exact test when the expected cell count was< 5. For comparisons of proportions between two independent groups reported in **Tables 1**–**3**, a two-sample Z-test for proportions based on the normal approximation was applied. A two-tailed P value< 0.05 was considered statistically significant.

**Table 1:** Distribution of glucose measurements across different glycemic ranges between the ELGM and control groups.

Blood glucose range	ELGM group (n = 14247)	Control group (n = 11559)	Z	P
Hypoglycemia				
severe	24 (0.17%)	13 (0.11%)	-1.182	0.237
mild	68 (0.48%)	37 (0.32%)	-1.973	**0.049**
Normal	8707 (61.11%)	5971 (51.66%)	-15.256	**<0.001**
Hyperglycemia				
level 1	3209 (22.52%)	2705 (23.40%)	1.668	0.095
level 2	1177 (8.26%)	1298 (11.23%)	8.052	**<0.001**
level 3	1062 (7.45%)	1535 (13.28%)	15.469	**<0.001**

Severe hypoglycemia (<3.0 mmol/L), Mild hypoglycemia (3.0 - 3.9 mmol/L), Normal glucose (3.9 - 10.0 mmol/L), Level 1 hyperglycemia (10.0 - 13.9 mmol/L), Level 2 hyperglycemia (13.9 - 16.7 mmol/L), Level 3 hyperglycemia (>16.7 mmol/L).Bold values indicate P < 0.05.

**Table 2:** Comparison of normal glucose achievement rates among patients with hyperglycemia.

Hyperglycemia category	ELGM group (n = 625)			Control group (n = 513)			Z	P
	Patients (n)	Measurements (n)	Normal glucose n (%)	Patients (n)	Measurements (n)	Normal glucose n (%)		
level 1	190	3149	2563 (81.39%)	147	2133	1728 (81.01%)	0.35	0.729
level 2	84	2086	1446 (69.32%)	67	1524	1026 (67.32%)	1.28	0.201
level 3	189	5861	2402 (40.98%)	170	6250	1966 (31.45%)	10.91	**<0.001**

Level 1 hyperglycemia (10.0 - 13.9 mmol/L), Level 2 hyperglycemia (13.9 - 16.7 mmol/L), Level 3 hyperglycemia (>16.7 mmol/L).Bold values indicate P < 0.05.

**Table 3:** Comparison of drug-induced hypoglycemia Incidence between the ELGM and control groups.

Hypoglycemia category	ELGM group (n = 625)		Control group (n = 513)		Z	P
	Patients (n)	Iatrogenic (n,%)	Patients (n)	Iatrogenic (n,%)		
Severe	18	4 (22.2)	6	0 (0%)	1.265	0.206
Mild	32	6 (18.8)	9	2 (22.2%)	-0.232	0.816

Severe hypoglycemia (<3.0 mmol/L), Mild hypoglycemia (3.0 - 3.9 mmol/L).

## 3 Results

### 3.1 Baseline characteristics

**Figure 1:**
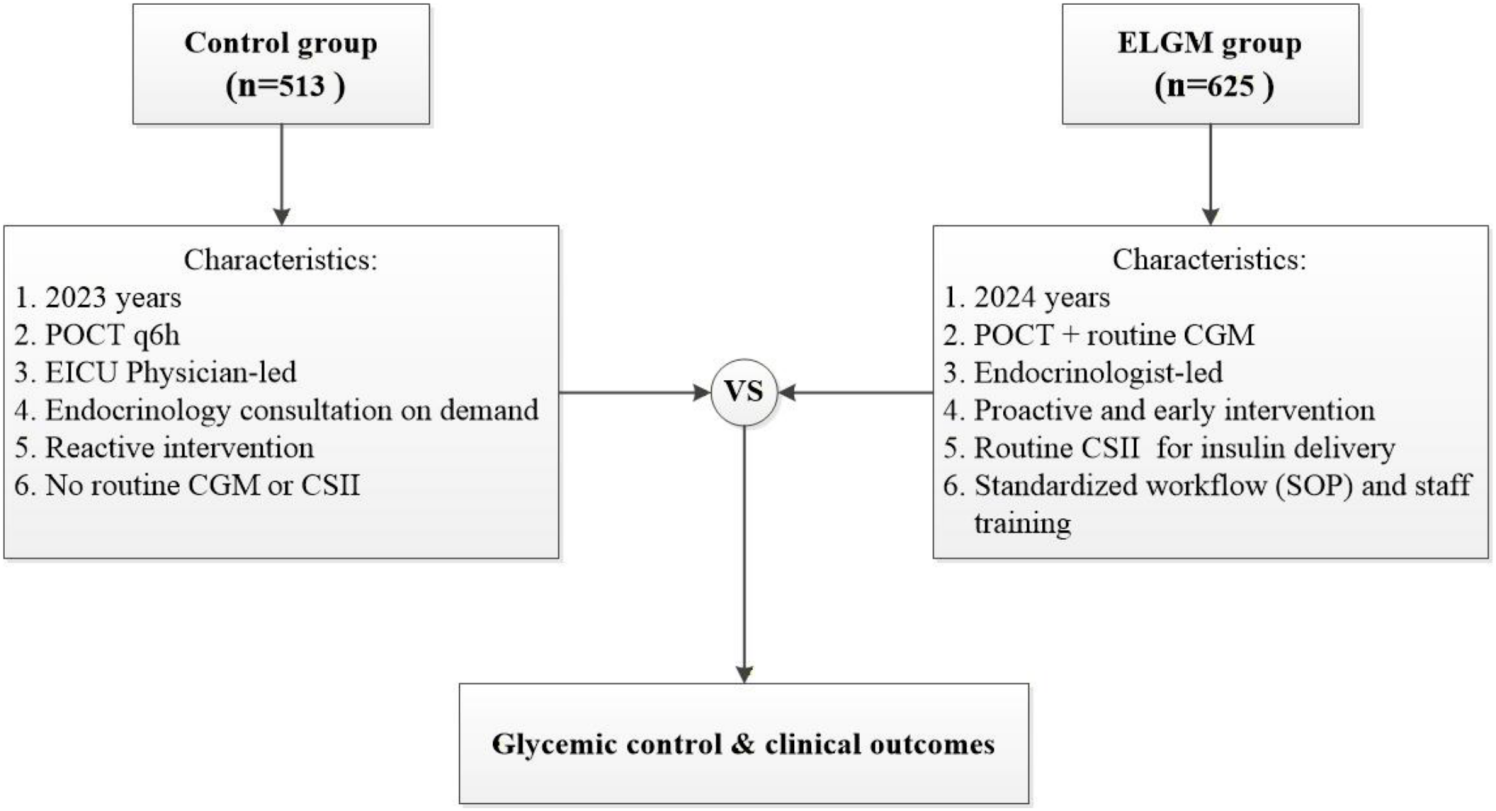
Flow chart of the study.

A total of 625 patients admitted to the EICU between January 1 and December 31, 2024 were enrolled in the ELGM group. The control group included 513 patients admitted between January 1 and December 31, 2023. The flow chart of the study was shown in **Figure 1**.

The baseline clinical characteristics of both groups are summarized in **Table 4**. No significant differences were observed in age, sex, glycated hemoglobin (HbA1c), glycated serum protein (GSP), history of diabetes, 24-hour SOFA score, or principal diseases, indicating good comparability between the two cohorts.

**Table 4:** Comparison of baseline data and GMS between the two groups of patients.

Variables	ELGM group (n = 625)	Control group (n = 513)	P
Age (years)	64.39 ± 16.38	63.07 ± 16.84	0.181
Sex (male)	370 (59.11%)	325 (63.35%)	0.144
Diabetes	138 (22.04%)	109 (21.25%)	0.745
Ketosis acidosis	10 (1.60%)	11 (2.14%)	0.495
24h-SOFA	5.35 ± 3.27	5.30 ± 3.47	0.785
HbA1c	6.22 ± 1.92	7.36 ± 2.31	0.239
GSP	2.56 ± 2.32	2.41 ± 0.65	0.348
Principal diseases			
cerebral hemorrhage	176 (28.16%)	146 (28.46%)	0.911
pneumonia	115 (18.40%)	110 (21.44%)	0.200
cerebral infarction	100 (16.00%)	77 (15.01%)	0.646
trauma	52 (8.32%)	38 (7.41%)	0.570
myocardial infarction	20 (3.20%)	16 (3.12%)	0.938
Others	162 (25.92%)	126 (24.56%)	0.600
GMS			
SC insulin	125 (20.00%)	167 (32.55%)	**<0.001**
IV insulin	8 (1.28%)	32 (6.24%)	**<0.001**
CSII	92 (14.72%)	0 (0%)	**<0.001**
Corticosteroid	148 (23.68%)	107 (20.86%)	0.256
CRRT	56 (8.96%)	40 (7.80%)	0.483
Nutritional strategies			
Short-peptide enteral	128 (20.48%)	71 (13.8%)	**0.003**
Whole-protein enteral	156 (24.96%)	100 (19.49%)	**0.028**
Diabetes-specific enteral	85 (13.6%)	60 (11.70%)	0.338

SOFA, sequential organ failure assessment; HbA1c, glycated hemoglobin; GSP, glycated serum protein; GMS, glucose management strategies; SC, subcutaneous injections; IV, intravenous infusion; CSII, Continuous subcutaneous insulin infusion; CRRT, continuous renal replacement therapy.Bold values indicate P < 0.05.

The proportions of patients receiving systemic corticosteroids and CRRT during the EICU stay were comparable between the ELGM and control groups, with no statistically significant differences observed. Regarding enteral nutrition strategies, the ELGM group showed a higher utilization of short-peptide enteral formulas (P = 0.003) and whole-protein enteral formulas (P = 0.028) compared with the control group, whereas the use of diabetes-specific enteral formulas did not differ significantly between the two groups. Furthermore, the overall application of enteral nutrition was greater in the ELGM group, which may have increased the complexity of glycemic management in this cohort.

Regarding glucose management strategies, the ELGM group adopted: Subcutaneous insulin injections in 125 patients (20.0%), including regimens based on multiple daily injections and regular insulin; Continuous subcutaneous insulin infusion (CSII) via insulin pumps in 92 patients (14.72%); Intravenous insulin infusion in 8 patients (1.28%). In contrast, the control group used: Subcutaneous insulin injections in 167 patients (32.55%); Intravenous insulin infusion in 32 patients (6.24%) (P< 0.001). This distribution reflects the structured insulin delivery strategies implemented under the ELGM management model, in which endocrinologists actively optimized insulin administration methods, including greater use of CSII, as part of the integrated glucose management workflow.

### 3.2 Comparison of overall glucose levels between groups

In the ELGM group, a total of 14,287 glucose measurements were recorded (mean 22.82 measurements per patient). After excluding 40 non-eligible measurements, 14,247 data points were included in the final analysis. In the control group, 12,189 glucose measurements were recorded (mean 23.76 per patient). After excluding 630 measurements, 11,559 data points were retained, representing 0.94 fewer valid measurements per patient compared with the ELGM group.

**Figure 2:**
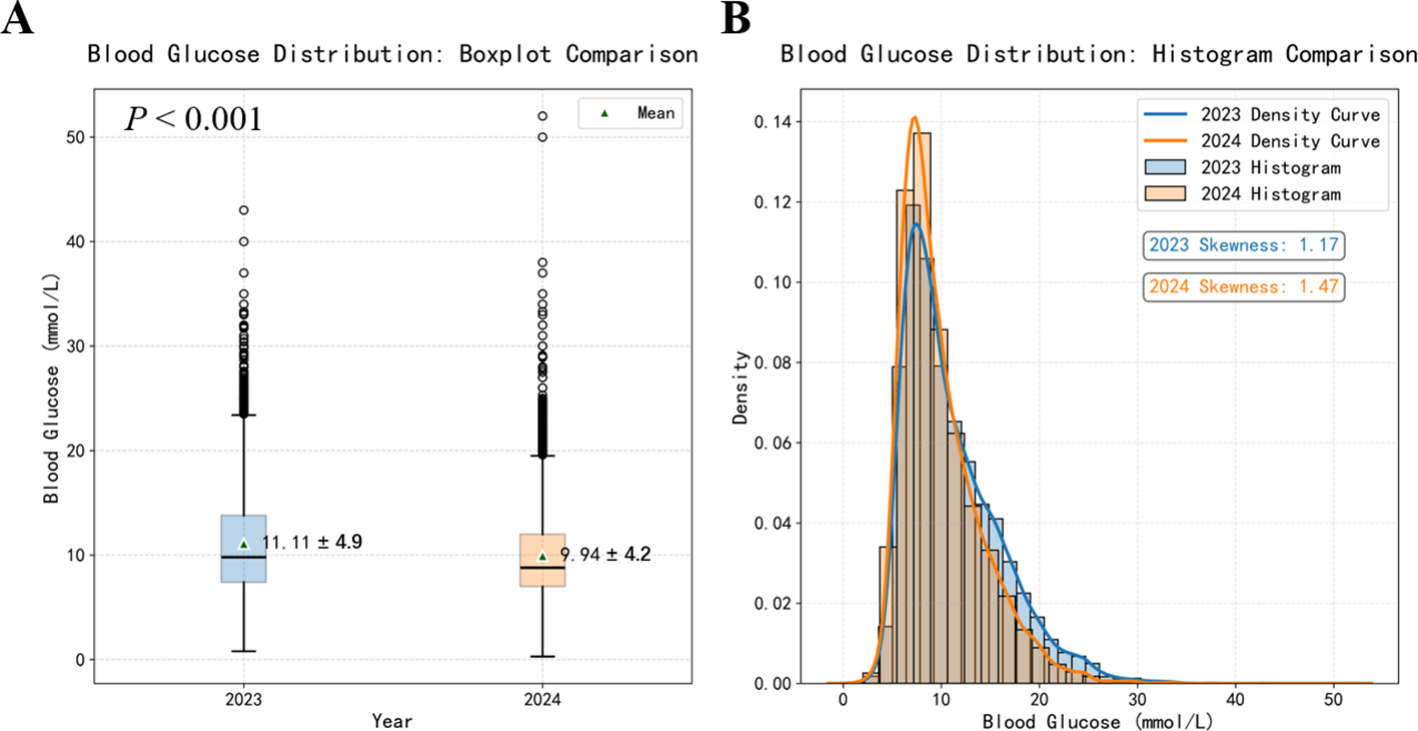
Comparison chart of overall blood glucose distribution. Panel **(A)** Box plot comparison of all blood glucose measurements in 2023 and 2024. Panel **(B)** Histogram and kernel density curve comparison of all blood glucose measurements in 2023 and 2024.

The comparison of overall glucose metrics between the two groups is presented in **Figure 2**. The mean glucose level in the ELGM group was significantly lower than that in the control group (9.9 vs. 11.1 mmol/L), representing a reduction of 1.2 mmol/L. Similarly, glucose variability, reflected by the standard deviation, decreased from 4.9 mmol/L in the control group to 4.2 mmol/L in the ELGM group, corresponding to a 14.3% reduction (P< 0.001).

### 3.3 Distribution of blood glucose intervals between the two groups

The comparative distribution of glucose levels between the ELGM and control groups is summarized in **Table 1**. The ELGM group demonstrated a significantly higher proportion of glucose measurements within the normal glucose range (3.9 - 10.0 mmol/L) compared with the control group (61.11% vs. 51.66%), representing an increase of 9.45% (P< 0.001).

The proportion of glucose measurements in the severe hypoglycemia range (<3.0 mmol/L) slightly increased from 0.11% to 0.17%, but the difference was not statistically significant (P = 0.237). Similarly, the mild hypoglycemia range (3.0 - 3.9 mmol/L) increased modestly from 0.32% to 0.48%, with borderline statistical significance (P = 0.049).

Conversely, the proportion of glucose measurements in the Level 1 hyperglycemia range (10.0 - 13.9 mmol/L) showed a slight, nonsignificant decrease (22.52% vs. 23.40%, P = 0.095). Notably, the proportions of glucose measurements in Level 2 (13.9 - 16.7 mmol/L) and Level 3 (>16.7 mmol/L) hyperglycemia significantly decreased in the ELGM group compared to the control group (8.26% vs. 11.23%, P< 0.001; 7.45% vs. 13.28%, P< 0.001, respectively).

Overall, the ELGM group exhibited a markedly improved glycemic profile, with a higher proportion of glucose measurements within the normal glucose range and significantly reduced proportions of measurements in both Level 2 and Level 3 hyperglycemic ranges. These results suggest that ELGM effectively enhanced overall glycemic control and reduced glucose variability.

### 3.4 Distribution of blood glucose among patients stratified by glycemic intervals

The distribution of blood glucose levels and target achievement rates among patients stratified by hyperglycemia intervals were summarized in **Table 2**.

In the Level 1 hyperglycemia group (10.0 - 13.9 mmol/L), the proportion of time within the normal glucose range was 81.39% (2563/3149) in the ELGM group and 81.01% (1728/2133) in the control group. The difference between the two groups was not statistically significant (P = 0.729). For patients in the Level 2 hyperglycemia range (13.9-16.7 mmol/L), the target glucose achievement rate was 69.32% (1446/2086) in the ELGM group and 67.32% (1026/1524) in the control group, showing no significant intergroup difference (P = 0.201). However, among Level 3 hyperglycemia patients (>16.7 mmol/L), the ELGM group exhibited a significantly higher target glucose achievement rate compared with the control group (40.98% [2402/5861] vs. 31.45% [1966/6250], P< 0.001). Overall, while there were no significant differences in glucose achievement rates between the ELGM and control groups in Level 1 and Level 2 hyperglycemia, the ELGM group demonstrated notably improved glycemic normalization in Level 3 hyperglycemia, suggesting that ELGM provides greater benefit for patients with severe hyperglycemia.

To further illustrate these differences, comparative boxplots and histograms of blood glucose levels among hyperglycemic patients were plotted (**Figures 2A**, **B**).

**Figure 3:**
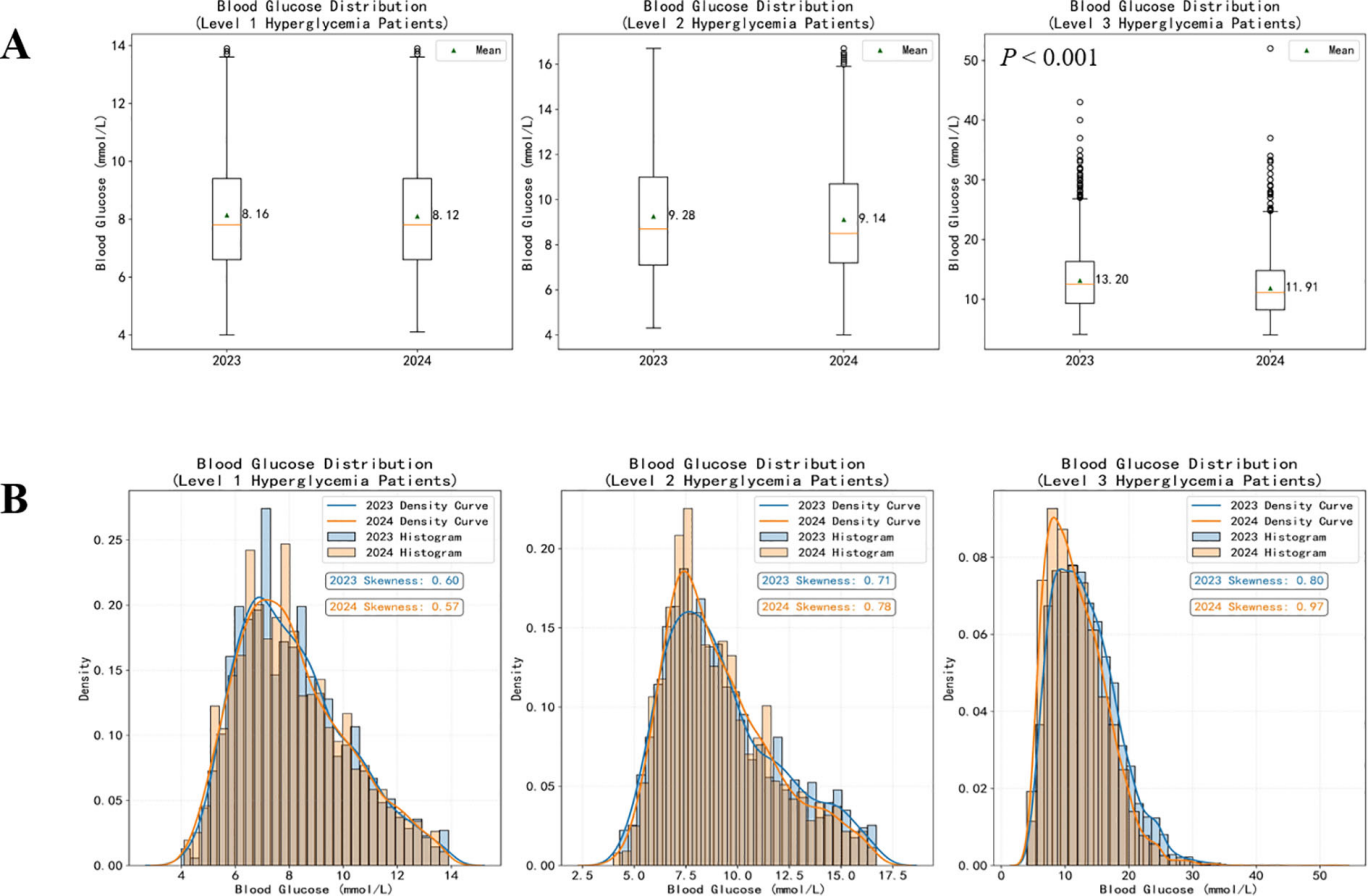
Box plot and histogram of blood glucose levels in patients with hyperglycemia. Panels **(A1–A3)** Box plot comparison of blood glucose levels in patients with Level 1, Level 2, and Level 3 hyperglycemia in 2023 and 2024, respectively. Panels **(B1–B3)** Histogram and kernel density curve comparison of blood glucose levels in patients with Level 1, Level 2, and Level 3 hyperglycemia in 2023 and 2024, respectively.

In the boxplots (**Figure 3A**), the ELGM group (2024) exhibited lower mean glucose values compared with the control group (2023), indicating an overall downward shift in glucose levels among hyperglycemic patients. The reduction in Level 3 hyperglycemia reached statistical significance (P< 0.001). The distribution plots (**Figure 3B**) further confirm this trend. In both Level 2 and Level 3 hyperglycemia, the ELGM group (2024) demonstrated glucose distributions that were more concentrated toward the normal range, with markedly fewer extremely high-glucose values. This phenomenon was especially pronounced in Level 3 hyperglycemia, where the histogram and density curve revealed increased skewness (0.97 vs. 0.80 in 2023), reflecting a shift of the overall distribution toward target glucose levels.

### 3.5 Analysis of patients in the hypoglycemic range

The incidence of drug-induced hypoglycemia is summarized in **Table 3**. In the ELGM group, 18 patients experienced severe hypoglycemia (<3.0 mmol/L), among whom 4 cases were identified as drug-induced hypoglycemia, including 2 cases during subcutaneous insulin pump therapy and 2 during intravenous insulin infusion. Additionally, 32 patients experienced mild hypoglycemia (3.0 - 3.9 mmol/L), with 6 cases classified as iatrogenic, including 3 during subcutaneous insulin pump use, 2 during intravenous insulin infusion, and 1 during subcutaneous insulin injection. In contrast, in the control group, 6 patients experienced severe hypoglycemia (<3.0 mmol/L), with no cases of drug-induced hypoglycemia. Nine patients developed mild hypoglycemia (3.0 - 3.9 mmol/L), among whom 2 cases were iatrogenic, both occurring during subcutaneous insulin injection.

Comparisons between the two groups using the Z test for proportions revealed no statistically significant differences in the incidence of drug-induced hypoglycemia for either severe (<3.0 mmol/L) or mild (3.0 - 3.9 mmol/L) hypoglycemia (P > 0.05 for both).

Overall, these results indicate that while hypoglycemic events occurred slightly more frequently in the ELGM group, the incidence of drug-induced hypoglycemia did not differ significantly between the ELGM and control groups, suggesting that ELGM did not increase the risk of treatment-induced hypoglycemia.

### 3.6 Clinical outcome indicators

Clinical outcomes are summarized in **Table 5**. No significant differences were observed between the ELGM and control groups in total hospital length of stay (16.76 ± 13.54 vs. 17.12 ± 20.14 days; P = 0.729), ICU length of stay (6.37 ± 6.98 vs. 6.54 ± 11.62 days; P = 0.771), or total hospitalization costs (¥86,312 ± 73,013 vs. ¥81,648 ± 98,302; P = 0.298). In the ELGM group, CGM was charged at ¥900 per 14 days and insulin pump therapy at ¥6 per hour. Based on the average duration of device use, these device-related costs represented only a small proportion of the overall hospitalization expenditure (approximately<1%). The duration of mechanical ventilation was modestly but significantly longer in the ELGM group (5.94 ± 6.23 vs. 5.15 ± 4.93 days; P = 0.017). Despite this, the ELGM group demonstrated a significantly lower incidence of nosocomial infections (15.02% vs. 27.68%; P< 0.001) and hyperglycemic hyperosmolar events (1.28% vs. 5.65%; P< 0.001).

**Table 5:** Comparison of clinical outcomes between the ELGM and control groups.

Variables	ELGM group (n=625)	Control group (n=513)	P
Length of stay (days)	16.76 ± 13.54	17.12 ± 20.14	0.729
EICU stay (days)	6.37 ± 6.98	6.54 ± 11.62	0.771
Ventilator use (days)	5.94 ± 6.23	5.15 ± 4.93	**0.017**
Total cost (CNY)	86312 ± 73013	81648 ± 98302	0.298
Hospital-acquired infection (n, %)	94 (15.02%)	142 (27.68%)	**<0.001**
Hyperglycemic hypersomolar (n, %)	8 (1.28%)	29 (5.65%)	**<0.001**

Bold values indicate P < 0.05.

## 4 Discussion

Hyperglycemia is common in critically ill patients, affecting up to 80% of ICU admissions (1, 23), and is associated with adverse outcomes including infection, organ dysfunction, and mortality. Effective management requires timely decision-making and specialized expertise, highlighting the need for structured, specialist-led approaches.

Prior to 2024, glycemic management in our EICU relied on a consultation-based model, in which EICU physicians served as the primary decision-makers and endocrinologists provided intermittent recommendations. This approach was frequently associated with delayed therapeutic adjustments, fragmented follow-up, and limited continuity of care (8, 9, 24). To address these limitations, we implemented an ELGM model. Compared with the conventional approach, ELGM not only increased the proportion of normoglycemia and reduced overall and severe hyperglycemia, but also decreased glycemic variability and demonstrated a trend toward lower rates of nosocomial infections.

Hypoglycemia, particularly severe episodes, is a major safety concern. In this study, ELGM was associated with a modest increase in overall hypoglycemia, but no significant rise in severe hypoglycemia (**Table 3**), and rates of drug-induced hypoglycemia were similar between groups, suggesting improved control without compromising safety. Glycemic variability is a key prognostic factor (29, 30). ELGM reduced mean glucose and variability (**Figure 1**), reflecting combined effects of endocrinologist-led decisions, CGM trend monitoring, CSII precision delivery, and optimized workflows. In this model, insulin delivery strategies were actively optimized by endocrinologists, including increased use of CSII, which facilitated more precise insulin titration and contributed to improved glycemic stability.

Point-of-care (POC) testing remains standard but is intermittent and increases nursing workload. In this study, CGM complemented POC, enabling earlier adjustments, supporting CSII use, reducing IV insulin reliance, and improving workflow efficiency. Recent ICU RCTs using CGM show heterogeneous results. Lu et al. (27) reported increased individual-level TIR (8–10 mmol/L) from 29% to 51.5% in critically ill patients, consistent with our departmental-level PGMR increase. Other studies [Holzinger (28), Boom (29), De Block (30), Franck (26)] found no significant TIR improvements, likely due to differences in baseline control, patient populations, and monitoring approaches. Several factors explain this heterogeneity. Our EICU cohort had high glucose variability, allowing ELGM to achieve meaningful improvement. Prior studies often used CGM passively, whereas here, POC guided treatment and CGM provided trend information, mitigating limitations of CGM accuracy in critical patients. Endocrinologists acted as primary decision-makers, providing continuous, individualized management, reducing delays and discontinuity inherent in consultation-based models.

ELGM also reduced infection rates, consistent with evidence linking hyperglycemia to infection risk (31–33). Improved departmental glycemic control may enhance immune function and reduce adverse events, without increasing overall hospitalization costs despite advanced monitoring and CSII use (24, 31, 33, 34).

This study has several strengths, including a large sample size and the implementation of a structured, ELGM model in routine practice. By integrating RT-CGM and insulin pump technologies into a coordinated workflow, ELGM enabled proactive, continuous glycemic management while maintaining patient safety and reducing nursing workload. However, as a single-center, retrospective before-after study comparing two calendar years, it is subject to temporal and contextual bias, and residual confounding cannot be excluded. Some clinically relevant variables, such as vasopressor use and total daily insulin dose, were unavailable for all patients, and glycemic outcomes were analyzed at the measurement rather than patients’ level, which may underestimate variance and limit causal inference. Finally, the inclusion of only EICU patients limits generalizability to other ICU or inpatient settings, and future multicenter, prospective studies with patient-level analyses are warranted to validate these findings.

In conclusion, in this retrospective EICU study, an endocrinologist-led glucose management model supported by RT-CGM and insulin pump technologies was associated with improved glycemic control, reduced glycemic variability, and acceptable safety profiles. These findings suggest a potential role for specialist-led, technology-assisted glycemic management strategies in critically ill populations.

## Data Availability

The original contributions presented in the study are included in the article/**Supplementary Material**. Further inquiries can be directed to the corresponding authors.
